# A peroxisome proliferator-activated receptor gamma (*PPARG*) polymorphism is associated with zoledronic acid-related osteonecrosis of the jaw in multiple myeloma patients: analysis by DMET microarray profiling

**DOI:** 10.1111/j.1365-2141.2011.08622.x

**Published:** 2011-04-26

**Authors:** Maria T Di Martino, Mariamena Arbitrio, Pietro H Guzzi, Emanuela Leone, Francesco Baudi, Eugenio Piro, Tullia Prantera, Iole Cucinotto, Teresa Calimeri, Marco Rossi, Pierangelo Veltri, Mario Cannataro, Pierosandro Tagliaferri, Pierfrancesco Tassone

**Affiliations:** 1 Medical Oncology Unit, Magna Graecia University and Tommaso Campanella Cancer Centre, Salvatore Venuta CampusCatanzaro; 2 Institute of Neurological Science-National Council of Research-UOS of Roccelletta di BorgiaCatanzaro; 3 Bioinformatics Laboratory, Magna Graecia UniversityCatanzaro; 4 Haematology Unit, Azienda Ospedaliera Pugliese-CiaccioCatanzaro; 5 Medical Oncology Unit, Ospedale San Giovanni di DioCrotone, Italy E-mail: tassone@unicz.it

**Keywords:** multiple myeloma, *PPARG*, osteonecrosis of the jaw, zoledronic acid, DMET

The aminobisphosphonate zoledronic acid (ZA) is the most important antiresorptive agent for the treatment of multiple myeloma (MM)-related bone disease (BD). Osteonecrosis of the jaw (ONJ) is an important complication of ZA-treated MM patients (Vannucchi *et al*, 2005; Filleul *et al*, 2010). So far, the mechanism of ONJ pathogenesis has not been clearly elucidated. Recently, a genetic susceptibility to ONJ has been suggested and a polymorphism of the cytochrome P450 *CYP2C8* has been associated with ZA-related ONJ in MM (Sarasquete *et al*, 2008).

To further investigate the genetic bases of ONJ, we genotyped in a case-control study a cohort of 19 MM patients treated with ZA who developed [nine cases, median age 66 years (range: 63–79)] or not [10 matched controls, median age 69 years (range: 63–84)] ONJ. We used the novel Affymetrix DMET™ plus platform (Affymetrix, Santa Clara, CA, USA), which interrogates 1936 genetic variations in 225 genes associated with phase I–II drug metabolism, disposition and transport (Deeken, 2009). The study protocol was approved by our University Hospital Bioethical Committee and informed consent was obtained from each patient. All patients received ZA according to the conventional dose and administration schedule; the ONJ group received 20 ± standard deviation (SD) 5·1 treatment courses and the control group underwent 15·1 ± 4·2 courses. MM patients were homogeneous on clinical and pathological characteristics at diagnosis and on their response to treatment. ONJ was diagnosed by clinical examination and imaging, including radiographs and/or computed tomography or magnetic resonance imaging. Peripheral blood was collected and used for DNA extraction. Genotypes were determined for each single nucleotide polymorphism (SNP) site of the 1931 of all interrogated SNPs and for the five Copy Number Variations (CNVs) included in DMET™ Plus GeneChip. Pharmacogenomic profiles were generated by Affymetrix DMET™ Console software®. Statistical analysis was performed by two-tailed Fisher’s exact test. No correction for multiple comparisons was performed. Results are therefore to be interpreted as hypothesis generating.

**Fig 1:**
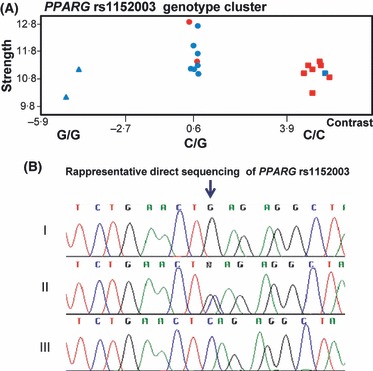
SNP rs1152003 genotype clustering of MM patients. (A) The red colour symbols are MM patients with ONJ. Blue colour symbols are matched MM control patients. Genotypes are identified as homozygote reference allele 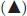, heterozygote (•) and homozygote variant allele (▪). (B) Representative electropherogram of direct sequencing analysis of a homozygote reference allele (I), heterozygote (II) and homozygote variant allele (III). The variant rs1152003 in PPARG gene was analyzed using the following PCR primers: Forward: 5′-TCCTTCTGCAAGGCAGTTTT-3′ and Reverse: 5′-CACGCCTTCAGGGAACTTAG-3′. The black arrow indicates the G, C or G/C sequences.

Eight SNPs were significantly (*P* ≤ 0·05) associated with ONJ occurrence. Table I shows these SNPs, the reference and variant allele and the genotype and allele frequencies. All alleles were in Hardy-Weinberg equilibrium. The four genes correlated to the eight statistically relevant SNPs were *PPARG* (peroxisome proliferator-activated receptor gamma), *ABP1*{amiloride binding protein 1 [amine oxidase (copper-containing)]}, *CHST11* [carbohydrate (chondroitin 4) sulfotransferase 11] and *CROT* (carnitine O-octanoyltransferase). The different distribution of SNP alleles and genotypes between ONJ patients and control cases are reported in Table II. The SNP rs1152003, mapping in *PPARG,* showed the strongest association with ONJ. We detected a highly significant (*P* = 0·0055) differential occurrence of the C/C homozygous (HOM) genotype in 77·7% of ONJ cases (7/9) *versus* only 10% of controls (1/10) (Fig 1A). Moreover, homozygous and heterozygous genotypes for the C variant were differently distributed between ONJ patients and the control group (Table II). The frequency of the C variant allele in the *PPARG* SNP underlines a highly significant association of the C allele with the ONJ group (*P* = 0·0064, Table II). No clinical association has been previously reported for these SNPs.

**Table I:** SNP polymorphisms associated with ONJ in MM patients.

Polymorphism Ref>Var	Effect	Ref. SNP alleles (A//B)	Genotype frequencies			Allele frequencies	
	
			Ref	HT	Var	A	B
*PPARG* rs 1152003	NA	C//G	8	9	2	0·658	0·342
*ABP1*_2357A>G	P545P	G//A	9	9	1	0·289	0·711
*ABP1*_4064G>A	S630S	G//A	9	9	1	0·711	0·289
*ABP1*_4107C>G	H645D	C//G	9	9	1	0·711	0·289
*CHST11*_> (rs2463437)	NA	G//A	7	11	1	0·342	0·658
*CHST11*_> (rs903247)	NA	C//T	7	10	2	0·368	0·632
*CHST11* rs2468110	NA	G//A	7	10	2	0·632	0·368
*CROT*_73879> (rs2097937)	NA	G//A	12	7	0	0·184	0·816

Distribution data for eight SNPs associated with ONJ in MM patients. Polymorphisms are reported as Reference (Ref), Heterozygosis (HT) and Variant (Var). Allele frequencies were calculated in the population included in the study using the Hardy-Weinberg equation.

**Table II:** Allele and genotype frequencies of polymorphisms in MM patients.

SNP and variants	Gene	Allele distribution		*P*	Genotype	Genotype distribution		*P*	Clinical association
	
		Cases	Controls			Cases (%)	Controls (%)		
rs 1152003									
C	*PPARG*	16/18	9/20	0·0064	CC	7/9 (77·7)	1/10 (10·0)	0·0055	Unknown
G		2/18	11/20		CG	2/9 (22·2)	7/10 (70·0)		
					GG	0/9 (00·0)	2/10 (20·0)		
rs10893									
G	*ABP1*	2/18	9/20	0·0327	AA	7/9 (77·7)	2/10 (20·0)	0·023	Unknown
A		16/18	11/20		AG	2/9 (22·2)	7/10 (70·0)		
					GG	0/9 (00·0)	1/10 (10·0)		
rs4725373									
G	*ABP1*	16/18	11/20	0·0327	GG	7/9 (77·7)	2/10 (20·0)	0·023	Unknown
A		2/18	9/20		AG	2/9 (22·2)	7/10 (70·0)		
					AA	0/9 (00·0)	1/10 (10·0)		
rs1049793									
C	*ABP1*	16/18	11/20	0·0327	CC	7/9 (77·7)	2/10 (20·0)	0·023	Unknown
G		2/18	9/20		CG	2/9 (22·2)	7/10 (70·0)		
					GG	0/9 (00·0)	1/10 (10·0)		
rs2463437									
G	*CHST11*	15/18	10/20	0·0434	AA	6/9 (66·6)	1/10 (10·0)	0·0198	Unknown
A		3/18	10/20		AG	3/9 (33·3)	8/10 (80·0)		
					GG	0/9 (00·0)	1/10 (10·0)		
rs903247									
C	*CHST11*	3/18	11/20	0·0205	TT	6/9 (66·6)	1/10 (10·0)	0·0198	Unknown
T		15/18	9/20		CT	3/9 (33·3)	7/10 (70·0)		
					CC	0/9 (00·0)	2/10 (20·0)		
rs2468110									
G	*CHST11*	14/18	10/20	0·1008	GG	6/9 (66·6)	1/10 (10·0)	0·0198	Unknown
A		4/18	10/20		AG	2/9 (22·2)	8/10 (80·0)		
					AA	1/9 (11·1)	1/10 (10·0)		
rs2097937									
G	*CROT*	6/18	1/20	0·0381	AG	6/9 (66·6)	1/10 (10·0)	0·0198	Unknown
A		12/18	19/20		AA	3/9 (33·3)	9/10 (90·0)		
					GG	0/9 (00·0)	0/10 (00·0)		

Distribution data for eight SNPs associated with ONJ in MM patients. Allele and genotype distribution between case and control groups. Polymorphisms are reported as rs number used in the human SNP database (http://www.ncbi.nlm.nih.gov/projects/SNP/). The *P* value was calculated by two-tailed Fisher’s exact test.

Direct nucleotide sequencing was carried out on patient specimens to further confirm the presence of genetic variations, using an Applied Biosystems ABI 3100 Genetic Analyser. We found a concordance rate of 100% between DMET genotyping and sequence analysis (Fig 1B).

The rs1152003 SNP maps in the 3′UTR region of *PPARG*, at position 12477055 of chromosome 3 (Genome Build 37.1). Although no clinical correlation has been reported for the rs1152003 variant, polymorphisms in *PPARG* have been associated with increased risk of a variety of diseases (Dallongeville *et al*, 2009). *PPARG* is located in the human chromosome 3, band 3p25. Chromosomal abnormalities, such as 3p deletion, have been identified in several hematologic malignancies. PPARG is involved in adipocyte differentiation and in angiogenesis (Rosen & Spiegelman, 2001). Recently, the PPARG pathway has been recognized as key mechanism for bone remodelling. It acts on mesenchymal stem cell differentiation by increasing adipogenesis but also inhibiting osteoblast and osteoclast formation. Moreover, *PPARG* polymorphisms correlate with the bone mass density (Ackert-Bicknell *et al*, 2008). However, a recent study on a wide cohort of Korean individuals, with idiopathic, steroid-induced or alcohol-induced osteonecrosis of the femoral head, failed to demonstrate a significant correlation with three common *PPARG* polymorphisms (Kim *et al*, 2007). Interestingly, modulation of PPARG activity within the bone marrow microenvironment has been recently shown to interfere with cytokines such as IL6, which is involved with a central role in the pathogenesis of MM (Wang *et al*, 2004), suggesting also that PPARG may represent a valuable therapeutic target in MM (Garcia-Bates *et al*, 2008).

The present study also showed that three SNPs identified in *ABP1* were associated with ONJ and were in linkage disequilibrium (data not shown). *ABP1* encodes a membrane glycoprotein that is expressed in many epithelial and haematopoietic tissues. Moreover, a further three ONJ-associated SNPs map to *CHST11*, which was recently described as a factor required for proper chondroitin sulfation and cartilage morphogenesis. Expression of the chondroitin sulfotransferase genes is crucial for the correct mammalian bone morphogenesis. Finally, the ONJ-associated rs2097937 maps to *CROT*, whose protein is involved in the trans-esterification of acyl-CoA molecules.

Our findings indicate that genetic polymorphisms are involved in the pathogenesis of ONJ in MM patients. The highly significant association of ONJ with the rs1152003 SNP polymorphism in *PPARG* strongly suggests this genetic variant as candidate biomarker for the identification of MM patients at risk of ONJ if treated with ZA. In fact, the C/C genotype demonstrated an odds ratio of 31·5 (95% confidence interval, 2·35–422·32) for developing ONJ following ZA treatment. Differently from the recent report (Sarasquete *et al*, 2008), where the study was based on the 500K Affymetrix high density array, we used the DMET platform that interrogates only highly selective SNPs associated with drug toxicity and has the advantage of avoiding an extremely high number of comparisons, which requires statistical corrections and large patient cohorts. We propose the rs1152003 C/C genotype as a candidate genetic biomarker for ONJ, which warrants validation in larger series.
